# 2-[7-Chloro-1,1-dioxo-2-(2,4,5-trifluoro­benz­yl)-3,4-dihydro-2*H*-1,2,4-benzothia­diazin-4-yl]acetic acid

**DOI:** 10.1107/S1600536812014468

**Published:** 2012-04-13

**Authors:** Yanchun Yang, Yuhua Guo, Changjin Zhu

**Affiliations:** a Department of Applied Chemistry, Beijing Institute of Technology, Zhongguancun South Street, 100081 Beijing, People’s Republic of China; b Department of Chemistry and Environmental Engineering, Anyang Institute of Technology, Henan 455000, People’s Republic of China

## Abstract

In the mol­ecule of the title compound, C_16_H_12_ClF_3_N_2_O_4_S, the thia­diazine ring adopts a half-chair conformation. The dihedral angle between the benzene ring of the benzothia­diazine ring system and trifluoro­phenyl group is 15.02 (7)°. In the crystal, centrosymmetrically related mol­ecules are linked into dimers *via* pairs of O—H⋯O hydrogen bonds, generating *R*_2_^2^(8) ring motifs. The dimers are further connected into a three-dimensional network by C—H⋯O hydrogen bonds.

## Related literature

For the pharmacological properties of benzothia­diazine derivatives, see: Longman & Hamilton (1992 ▶); Buckheit *et al.* (1994 ▶); Yamada & Tang (1993 ▶); Phillips *et al.* (2002 ▶); Braghiroli *et al.* (2002 ▶); Pirotte *et al.* (1998 ▶); Francotte *et al.* (2007 ▶). For the biological properties and synthetic details of the title compound, see: Chen *et al.* (2010 ▶).
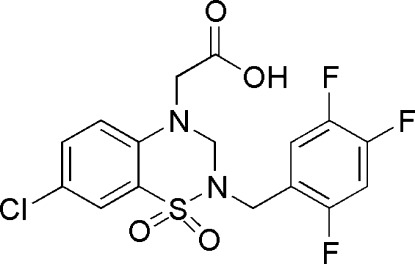


## Experimental

### 

#### Crystal data

C_16_H_12_ClF_3_N_2_O_4_S
*M* *_r_* = 420.79
Monoclinic, 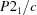

*a* = 9.3628 (2) Å
*b* = 12.3134 (2) Å
*c* = 15.5597 (3) Å
β = 105.996 (1)°
*V* = 1724.39 (6) Å^3^
*Z* = 4
Mo *K*α radiation
μ = 0.40 mm^−1^
*T* = 296 K
0.20 × 0.20 × 0.20 mm

#### Data collection

Bruker APEXII CCD diffractometer
15140 measured reflections
4293 independent reflections
3302 reflections with *I* > 2σ(*I*)
*R*_int_ = 0.027

#### Refinement

*R*[*F* ^2^ > 2σ(*F* ^2^)] = 0.047
*wR*(*F* ^2^) = 0.131
*S* = 1.04
4293 reflections
245 parameters
H-atom parameters constrained
Δρ_max_ = 0.73 e Å^−3^
Δρ_min_ = −0.82 e Å^−3^

### 

Data collection: *APEX2* (Bruker, 2005 ▶); cell refinement: *SAINT-Plus* (Bruker, 2001 ▶); data reduction: *SAINT-Plus*; program(s) used to solve structure: *SHELXS97* (Sheldrick, 2008 ▶); program(s) used to refine structure: *SHELXL97* (Sheldrick, 2008 ▶); molecular graphics: *SHELXTL* (Sheldrick, 2008 ▶); software used to prepare material for publication: *SHELXTL*.

## Supplementary Material

Crystal structure: contains datablock(s) I, global. DOI: 10.1107/S1600536812014468/rz2722sup1.cif

Structure factors: contains datablock(s) I. DOI: 10.1107/S1600536812014468/rz2722Isup2.hkl

Supplementary material file. DOI: 10.1107/S1600536812014468/rz2722Isup3.cml

Additional supplementary materials:  crystallographic information; 3D view; checkCIF report

## Figures and Tables

**Table 1:** Hydrogen-bond geometry (Å, °)

*D*—H⋯*A*	*D*—H	H⋯*A*	*D*⋯*A*	*D*—H⋯*A*
O4—H4⋯O3^i^	0.82	1.86	2.676 (2)	171
C3—H3⋯O1^ii^	0.93	2.47	3.391 (3)	169
C16—H16⋯O2^iii^	0.93	2.46	3.387 (2)	172
C13—H13⋯O3^iv^	0.93	2.51	3.307 (3)	144

Symmetry codes: (i) 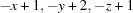; (ii) 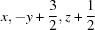; (iii) 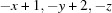; (iv) 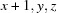.

**Fig. 1.**
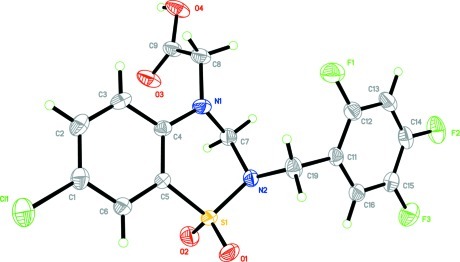
The molecular structure of the title compound, with 30% probability displacement ellipsoids.

## supplementary crystallographic information

### Comment

Benzothiadiazine derivatives have attracted considerable attention because they are endowed with a large spectrum of properties. Since the 1950's, various pharmacological investigations of newly synthesized benzothiadiazines demonstrated interesting pharmacological activities and showed great potential for the development of new medications for treating diseases (Longman & Hamilton, 1992; Buckheit *et al.*, 1994; Yamada & Tang, 1993; Phillips *et al.*, 2002; Braghiroli *et al.*, 2002; Pirotte *et al.*, 1998; Francotte *et al.*, 2007). The title compound, whose structure is reported herein, was synthesized and used as an aldose reductase inhibitor (Chen *et al.*, 2010).

In the molecule of the title compound (Fig. 1), the thiadiazine ring (C7/N1/C4/C5/S1/N2) adopts a half-chair conformation, with puckering parameters *Q*_T_ = 0.5164 (19) Å, θ = 47.5 (2)° and φ = -25.9 (3)°. The deviation of the S1, N1 and N2 atoms from the plane of the benzene ring of the benzothiadiazin ring system are -0.0726 (6), 0.0305 (19) and 0.2655 (18) Å, respectively. The dihedral angle formed by the two six-membered aromatic rings C1–C6 and C11–C16 is 15.02 (7)°. In the crystal, centrosymmetrically related molecules interact to form dimers through pairs of O—H···O hydrogen bonds (Table 1) generating a *R*^2^_2_(8) ring motif. The dimers are further linked into a three-dimensional network by intermolecular C—H···O hydrogen bonds (Table 1).

### Experimental

A mixture of methyl 2-(7-chloro-1,1-dioxido-2-(2,4,5-trifluorobenzyl)-2*H*-benzo[*e*][1,2,4]thiadiazin-4(3*H*)-yl)acetate (1 mmol), 1,4-dioxane (5 ml) and saturated aqueous sodium hydroxide (5 ml) was stirred at room temperature for 2 h. The alkaline suspension was adjusted to be acidic with 0.1 molar HCl and extracted with ethyl acetate (3 × 20 ml). The combined organic layers were dried over MgSO_4_ and filtered. The filtrate was concentrated to dryness under reduced pressure. Crystals suitable for X-ray diffraction were obtained by slow evaporation of a methanol solution of the compound (yield: 76%).

### Refinement

H atoms were positioned geometrically and refined using a riding model, with C—H = 0.93–0.97 Å, O—H = 0.82 Å and with *U*_iso_(H) = 1.2*U*_eq_(C) or 1.5*U*_eq_(O).

### Crystal data

**Table:**

C_16_H_12_ClF_3_N_2_O_4_S	*F*(000) = 856
*M**_r_* = 420.79	*D*_x_ = 1.621 Mg m^−^^3^
Monoclinic, *P*2_1_/*c*	Mo *K*α radiation, λ = 0.71073 Å
Hall symbol: -P 2ybc	Cell parameters from 4599 reflections
*a* = 9.3628 (2) Å	θ = 2.8–28.3°
*b* = 12.3134 (2) Å	µ = 0.40 mm^−^^1^
*c* = 15.5597 (3) Å	*T* = 296 K
β = 105.996 (1)°	Block, colourless
*V* = 1724.39 (6) Å^3^	0.20 × 0.20 × 0.20 mm
*Z* = 4	

### Data collection

**Table:**

Bruker APEXII CCD diffractometer	3302 reflections with *I* > 2σ(*I*)
Radiation source: fine-focus sealed tube	*R*_int_ = 0.027
Graphite monochromator	θ_max_ = 28.3°, θ_min_ = 2.1°
φ and ω scans	*h* = −12→12
15140 measured reflections	*k* = −16→15
4293 independent reflections	*l* = −20→20

### Refinement

**Table:**

Refinement on *F*^2^	Primary atom site location: structure-invariant direct methods
Least-squares matrix: full	Secondary atom site location: difference Fourier map
*R*[*F*^2^ > 2σ(*F*^2^)] = 0.047	Hydrogen site location: inferred from neighbouring sites
*wR*(*F*^2^) = 0.131	H-atom parameters constrained
*S* = 1.04	*w* = 1/[σ^2^(*F*_o_^2^) + (0.0571*P*)^2^ + 1.0199*P*] where *P* = (*F*_o_^2^ + 2*F*_c_^2^)/3
4293 reflections	(Δ/σ)_max_ < 0.001
245 parameters	Δρ_max_ = 0.73 e Å^−^^3^
0 restraints	Δρ_min_ = −0.82 e Å^−^^3^

### Special details

**Table:**

Geometry. All e.s.d.'s (except the e.s.d. in the dihedral angle between two l.s. planes) are estimated using the full covariance matrix. The cell e.s.d.'s are taken into account individually in the estimation of e.s.d.'s in distances, angles and torsion angles; correlations between e.s.d.'s in cell parameters are only used when they are defined by crystal symmetry. An approximate (isotropic) treatment of cell e.s.d.'s is used for estimating e.s.d.'s involving l.s. planes.
Refinement. Refinement of *F*^2^ against ALL reflections. The weighted *R*-factor *wR* and goodness of fit *S* are based on *F*^2^, conventional *R*-factors *R* are based on *F*, with *F* set to zero for negative *F*^2^. The threshold expression of *F*^2^ > σ(*F*^2^) is used only for calculating *R*-factors(gt) *etc*. and is not relevant to the choice of reflections for refinement. *R*-factors based on *F*^2^ are statistically about twice as large as those based on *F*, and *R*- factors based on ALL data will be even larger.

### Fractional atomic coordinates and isotropic or equivalent isotropic displacement parameters (Å^2^)

**Table:**

	*x*	*y*	*z*	*U*_iso_*/*U*_eq_	
S1	0.45256 (6)	0.88685 (5)	0.08722 (3)	0.04077 (16)	
Cl1	−0.00328 (10)	0.62773 (8)	0.09172 (6)	0.0872 (3)	
C11	0.8849 (2)	0.89923 (18)	0.16523 (14)	0.0394 (5)	
C5	0.3729 (2)	0.79827 (17)	0.14930 (12)	0.0350 (4)	
N2	0.62050 (19)	0.90503 (15)	0.15259 (11)	0.0387 (4)	
F2	1.26561 (18)	1.08588 (15)	0.23239 (13)	0.0768 (5)	
O1	0.46125 (19)	0.83390 (17)	0.00742 (10)	0.0607 (5)	
F3	1.0586 (2)	1.12091 (14)	0.07690 (12)	0.0729 (5)	
C12	0.9947 (3)	0.88393 (19)	0.24347 (15)	0.0458 (5)	
C6	0.2388 (2)	0.75123 (19)	0.10327 (14)	0.0449 (5)	
H6	0.1985	0.7648	0.0426	0.054*	
N1	0.5746 (2)	0.82433 (15)	0.28393 (11)	0.0392 (4)	
F1	0.97529 (19)	0.80656 (14)	0.30117 (11)	0.0714 (5)	
O2	0.37472 (19)	0.98763 (15)	0.07985 (13)	0.0597 (5)	
C4	0.4382 (2)	0.77835 (16)	0.24064 (12)	0.0341 (4)	
C7	0.6157 (2)	0.92240 (18)	0.24459 (13)	0.0400 (5)	
H7A	0.7126	0.9466	0.2802	0.048*	
H7B	0.5445	0.9792	0.2456	0.048*	
C3	0.3601 (3)	0.70893 (18)	0.28347 (14)	0.0435 (5)	
H3	0.3996	0.6932	0.3438	0.052*	
C2	0.2264 (3)	0.66393 (19)	0.23789 (16)	0.0484 (5)	
H2	0.1761	0.6192	0.2679	0.058*	
C1	0.1662 (3)	0.6843 (2)	0.14823 (16)	0.0489 (5)	
C9	0.5751 (3)	0.8936 (2)	0.43292 (13)	0.0447 (5)	
C8	0.6382 (3)	0.8131 (2)	0.37949 (13)	0.0462 (5)	
H8A	0.7449	0.8232	0.3935	0.055*	
H8B	0.6200	0.7400	0.3972	0.055*	
C19	0.7430 (2)	0.8351 (2)	0.14144 (16)	0.0468 (5)	
H19A	0.7213	0.8102	0.0800	0.056*	
H19B	0.7531	0.7720	0.1800	0.056*	
C16	0.9087 (2)	0.98074 (19)	0.10813 (15)	0.0442 (5)	
H16	0.8376	0.9939	0.0542	0.053*	
C15	1.0367 (3)	1.0414 (2)	0.13140 (16)	0.0482 (5)	
C14	1.1431 (3)	1.0230 (2)	0.21047 (18)	0.0506 (6)	
C13	1.1245 (3)	0.9440 (2)	0.26747 (17)	0.0527 (6)	
H13	1.1968	0.9307	0.3209	0.063*	
O4	0.6474 (2)	0.89659 (17)	0.51717 (10)	0.0651 (6)	
H4	0.6053	0.9380	0.5435	0.098*	
O3	0.4682 (2)	0.95079 (17)	0.39943 (11)	0.0645 (6)	

### Atomic displacement parameters (Å^2^)

**Table:**

	*U*^11^	*U*^22^	*U*^33^	*U*^12^	*U*^13^	*U*^23^
S1	0.0349 (3)	0.0559 (3)	0.0303 (2)	−0.0079 (2)	0.00703 (18)	0.0092 (2)
Cl1	0.0666 (5)	0.1026 (7)	0.0868 (6)	−0.0466 (5)	0.0117 (4)	0.0042 (5)
C11	0.0321 (10)	0.0454 (12)	0.0418 (11)	0.0012 (9)	0.0122 (8)	−0.0088 (9)
C5	0.0337 (10)	0.0405 (11)	0.0317 (9)	−0.0010 (8)	0.0107 (7)	0.0047 (8)
N2	0.0305 (8)	0.0499 (10)	0.0360 (8)	−0.0045 (7)	0.0095 (7)	−0.0032 (7)
F2	0.0449 (9)	0.0762 (11)	0.1085 (14)	−0.0195 (8)	0.0196 (9)	−0.0269 (10)
O1	0.0554 (10)	0.0992 (15)	0.0291 (7)	−0.0253 (10)	0.0143 (7)	−0.0050 (8)
F3	0.0804 (12)	0.0682 (11)	0.0810 (11)	−0.0100 (9)	0.0403 (10)	0.0087 (8)
C12	0.0422 (12)	0.0479 (13)	0.0464 (12)	0.0040 (10)	0.0108 (9)	0.0000 (10)
C6	0.0404 (12)	0.0546 (14)	0.0376 (10)	−0.0088 (10)	0.0072 (9)	0.0050 (9)
N1	0.0435 (10)	0.0441 (10)	0.0272 (8)	0.0020 (8)	0.0052 (7)	−0.0012 (7)
F1	0.0715 (11)	0.0740 (11)	0.0638 (10)	0.0000 (9)	0.0101 (8)	0.0207 (8)
O2	0.0453 (10)	0.0598 (11)	0.0706 (11)	0.0027 (8)	0.0101 (8)	0.0289 (9)
C4	0.0403 (11)	0.0337 (10)	0.0292 (9)	0.0057 (8)	0.0110 (8)	0.0000 (7)
C7	0.0384 (11)	0.0444 (12)	0.0362 (10)	−0.0029 (9)	0.0084 (8)	−0.0067 (8)
C3	0.0597 (14)	0.0408 (12)	0.0341 (10)	0.0057 (10)	0.0195 (9)	0.0073 (8)
C2	0.0582 (14)	0.0397 (12)	0.0555 (13)	−0.0028 (11)	0.0295 (11)	0.0075 (10)
C1	0.0440 (13)	0.0486 (13)	0.0552 (13)	−0.0125 (10)	0.0154 (10)	0.0009 (10)
C9	0.0438 (12)	0.0551 (14)	0.0305 (9)	0.0077 (10)	0.0025 (8)	−0.0027 (9)
C8	0.0468 (12)	0.0566 (14)	0.0310 (10)	0.0132 (11)	0.0036 (8)	−0.0007 (9)
C19	0.0383 (12)	0.0501 (13)	0.0527 (13)	−0.0044 (10)	0.0138 (9)	−0.0133 (10)
C16	0.0386 (11)	0.0549 (14)	0.0402 (11)	0.0035 (10)	0.0124 (9)	−0.0059 (9)
C15	0.0477 (13)	0.0486 (13)	0.0544 (13)	0.0009 (10)	0.0243 (11)	−0.0043 (10)
C14	0.0335 (11)	0.0531 (14)	0.0666 (15)	−0.0051 (10)	0.0160 (10)	−0.0196 (12)
C13	0.0352 (12)	0.0634 (16)	0.0525 (13)	0.0073 (11)	0.0005 (9)	−0.0104 (12)
O4	0.0648 (12)	0.0852 (14)	0.0333 (8)	0.0296 (10)	−0.0066 (8)	−0.0137 (8)
O3	0.0606 (11)	0.0843 (14)	0.0379 (8)	0.0325 (10)	−0.0042 (7)	−0.0133 (8)

### Geometric parameters (Å, º)

**Table:**

S1—O1	1.4245 (17)	C4—C3	1.407 (3)
S1—O2	1.4277 (19)	C7—H7A	0.9700
S1—N2	1.6357 (18)	C7—H7B	0.9700
S1—C5	1.7540 (19)	C3—C2	1.374 (3)
Cl1—C1	1.734 (2)	C3—H3	0.9300
C11—C12	1.373 (3)	C2—C1	1.376 (3)
C11—C16	1.398 (3)	C2—H2	0.9300
C11—C19	1.502 (3)	C9—O3	1.217 (3)
C5—C6	1.389 (3)	C9—O4	1.300 (2)
C5—C4	1.405 (3)	C9—C8	1.514 (3)
N2—C7	1.460 (2)	C8—H8A	0.9700
N2—C19	1.482 (3)	C8—H8B	0.9700
F2—C14	1.347 (3)	C19—H19A	0.9700
F3—C15	1.347 (3)	C19—H19B	0.9700
C12—F1	1.355 (3)	C16—C15	1.374 (3)
C12—C13	1.383 (3)	C16—H16	0.9300
C6—C1	1.376 (3)	C15—C14	1.372 (4)
C6—H6	0.9300	C14—C13	1.360 (4)
N1—C4	1.389 (3)	C13—H13	0.9300
N1—C8	1.448 (2)	O4—H4	0.8200
N1—C7	1.453 (3)		
			
O1—S1—O2	118.58 (12)	C4—C3—H3	119.4
O1—S1—N2	109.19 (10)	C3—C2—C1	120.7 (2)
O2—S1—N2	108.23 (11)	C3—C2—H2	119.7
O1—S1—C5	109.30 (11)	C1—C2—H2	119.7
O2—S1—C5	107.43 (10)	C6—C1—C2	120.2 (2)
N2—S1—C5	102.98 (9)	C6—C1—Cl1	119.63 (19)
C12—C11—C16	116.8 (2)	C2—C1—Cl1	120.18 (18)
C12—C11—C19	122.8 (2)	O3—C9—O4	123.6 (2)
C16—C11—C19	120.4 (2)	O3—C9—C8	122.87 (19)
C6—C5—C4	121.91 (19)	O4—C9—C8	113.50 (19)
C6—C5—S1	115.88 (15)	N1—C8—C9	112.90 (18)
C4—C5—S1	122.18 (16)	N1—C8—H8A	109.0
C7—N2—C19	115.69 (18)	C9—C8—H8A	109.0
C7—N2—S1	110.23 (13)	N1—C8—H8B	109.0
C19—N2—S1	119.35 (14)	C9—C8—H8B	109.0
F1—C12—C11	118.7 (2)	H8A—C8—H8B	107.8
F1—C12—C13	117.7 (2)	N2—C19—C11	109.07 (18)
C11—C12—C13	123.6 (2)	N2—C19—H19A	109.9
C1—C6—C5	119.4 (2)	C11—C19—H19A	109.9
C1—C6—H6	120.3	N2—C19—H19B	109.9
C5—C6—H6	120.3	C11—C19—H19B	109.9
C4—N1—C8	121.46 (18)	H19A—C19—H19B	108.3
C4—N1—C7	116.70 (16)	C15—C16—C11	120.2 (2)
C8—N1—C7	115.48 (18)	C15—C16—H16	119.9
N1—C4—C5	120.34 (18)	C11—C16—H16	119.9
N1—C4—C3	123.12 (18)	F3—C15—C14	119.1 (2)
C5—C4—C3	116.53 (19)	F3—C15—C16	120.2 (2)
N1—C7—N2	112.00 (17)	C14—C15—C16	120.8 (2)
N1—C7—H7A	109.2	F2—C14—C13	120.0 (2)
N2—C7—H7A	109.2	F2—C14—C15	119.3 (2)
N1—C7—H7B	109.2	C13—C14—C15	120.7 (2)
N2—C7—H7B	109.2	C14—C13—C12	117.9 (2)
H7A—C7—H7B	107.9	C14—C13—H13	121.1
C2—C3—C4	121.29 (19)	C12—C13—H13	121.1
C2—C3—H3	119.4	C9—O4—H4	109.5

### Hydrogen-bond geometry (Å, º)

**Table:**

*D*—H···*A*	*D*—H	H···*A*	*D*···*A*	*D*—H···*A*
O4—H4···O3^i^	0.82	1.86	2.676 (2)	171
C3—H3···O1^ii^	0.93	2.47	3.391 (3)	169
C16—H16···O2^iii^	0.93	2.46	3.387 (2)	172
C13—H13···O3^iv^	0.93	2.51	3.307 (3)	144

Symmetry codes: (i) −*x*+1, −*y*+2, −*z*+1; (ii) *x*, −*y*+3/2, *z*+1/2; (iii) −*x*+1, −*y*+2, −*z*; (iv) *x*+1, *y*, *z*.

## References

[bb1] Braghiroli, D., Puia, G., Cannazza, G., Tait, A., Parenti, C., Losi, G. & Baraldi, M. (2002). *J. Med. Chem.* **45**, 2355–2357. 10.1021/jm025510d 12036344

[bb2] Bruker (2001). *SAINT-Plus* Bruker AXS Inc., Madison, Wisconsin, USA.

[bb3] Bruker (2005). *APEX2* . Bruker AXS Inc., Madison, Wisconsin, USA.

[bb4] Buckheit, R. W. Jr, Fliakas-Boltz, V., Decker, W. D., Roberson, J. L., Pyle, C. A., White, E. L., Bowdon, B. J., McMahon, J. B., Boyd, M. R., Bader, J. P., Nickell, D. G., Barth, H. & Antonucci, T. K. (1994). *Antivir. Res.* **25**, 43–56. 10.1016/0166-3542(94)90092-2 7529014

[bb5] Chen, X., Zhu, C. J., Guo, F., Qiu, X. W., Yang, Y. C., Zhang, S. Z., He, M. L., Parveen, S., Jing, C. J., Li, Y. & Ma, B. (2010). *J. Med. Chem.* **53**, 8330–8344. 10.1021/jm100962a 21062005

[bb6] Francotte, P., de Tullio, P., Goffin, E., Dintilhac, G., Graindorge, E., Fraikin, P., Lestage, P., Danober, L., Thomas, J.-Y., Caignard, D.-H. & Pirotte, B. (2007). *J. Med. Chem.* **50**, 3153–3157. 10.1021/jm070120i 17552506

[bb7] Longman, S. D. & Hamilton, T. C. (1992). *Med. Res. Rev.* **12**, 73–148. 10.1002/med.2610120202 1535674

[bb8] Phillips, D., Sonnenberg, J., Arai, A. C., Vaswani, R., Krutzik, P. O., Kleisli, T., Kessler, M., Granger, R., Lynch, G. & Chamberlin, A. R. (2002). *Bioorg. Med. Chem.* **10**, 1229–1248. 10.1016/s0968-0896(01)00405-9 11886787

[bb9] Pirotte, B., Podona, T., Diouf, O., de Tullio, P., Lebrun, P., Dupont, L., Somers, F., Delarge, J., Morain, P., Lestage, P., Lepagnol, J. & Spedding, M. (1998). *J. Med. Chem.* **41**, 2946–2959. 10.1021/jm970694v 9685234

[bb10] Sheldrick, G. M. (2008). *Acta Cryst.* A**64**, 112–122. 10.1107/S0108767307043930 18156677

[bb11] Yamada, K. A. & Tang, C. M. (1993). *J. Neurosci.* **13**, 3904–3915. 10.1523/JNEUROSCI.13-09-03904.1993 PMC6576449 8103555

